# Forecasting Alzheimer’s disease progression using non-imaging clinical features and XGBoost

**DOI:** 10.64898/2026.10.05.26364229

**Published:** 2026-10-07

**Authors:** Andrew Gautier, Lingjian Zheng, Bernard G. Schreurs

## Abstract

Identifying progressive dementia during its prodromal stage is a challenging yet critical task for healthcare providers. Traditional clinical assessments are often not sensitive enough to detect warning signs of cognitive decline. Machine learning (ML) methods have shown promise in modeling clinical trajectories associated with neurodegenerative disorders such as Alzheimer’s disease (AD). Validation of these models’ forecasting abilities, however, is widely lacking in the literature. To address this gap, we established a longitudinal cohort from the National Alzheimer’s Coordinating Center ( *n* = 15,727) comprising individuals who were cognitively normal (CN) or had mild cognitive impairment (MCI) at baseline. Cognitive status and clinical observations collected over up to 10 visits were used to train several ML classifiers. We employed the gradient-boosted tree ensemble method XGBoost with a custom feature-engineering pipeline. On non-imputed data, our models achieved an AUC-ROC of 0.9447 [0.9417–0.9476] and PR-AUC of 0.8554 [0.8497–0.8611] for the CN→MCI task, and an AUC-ROC of 0.9059 [0.8983–0.9134] and PR-AUC of 0.9214 [0.9156–0.9272] for the MCI→AD task. In a lead-time forecasting analysis spanning mean follow-up periods of 56.1 months (range, 9.1–214.1) for CN→MCI and 42.7 months (range, 9.1–126.1) for MCI→AD, the non-imputed models detected future progression before diagnosis in 29.58% [28.29%–30.87%] of CN→MCI progressors and 22.41% [21.36%–23.46%] of MCI→AD progressors. Among these individuals, the mean lead time was 33.04 months [32.52–33.56] and 17.95 months [17.71–18.19], respectively. False alarm rates among non-progressors were 12.6% [11.5%–13.7%] and 20.0% [18.4%–21.6%], respectively. These findings suggest that longitudinal machine learning models can identify future cognitive decline before diagnosis, indicating clinical utility for early dementia risk stratification.

**Author summary:**

Alzheimer’s disease (AD) is a progressive and currently incurable neurodegenerative disorder that remains highly difficult to diagnose during its early stages. While modern early-stage dementia research relies on advanced neuroimaging, the high cost of these tests excludes many vulnerable communities. We designed our study to advance scalable, low-cost diagnostic alternatives that can identify individuals at significant risk of cognitive decline, ensuring faster access to emerging therapies.

We examined trajectories of cognitive decline in 15,727 adults (mean age = 71.45 years, SD = 9.06) who had between 2 and 10 follow-up visits. Using 37 demographic, behavioral, clinical, and genetic variables, we modeled cognitively stable and declining trajectories using the XGBoost algorithm. Additionally, we evaluated the models’ ability to forecast future diagnoses and the impact of longitudinal imputation.

Our models performed well in both classification tasks (AUC-ROC ≥ 0.90).

Imputation did not substantively alter the classifiers’ performance. In forecasting diagnosis conversion, the models raised alarms before the recorded conversion visit in a subset of progressors, with non-imputed models yielding longer net lead times than imputed models in AD forecasting. This work describes a strategy for handling mixed-length temporal dimensions in longitudinal cohorts and evaluates its prognostic ability.

## Full Text Availability

The license terms selected by the author(s) for this preprint version do not permit archiving in PMC. The full text is available from the preprint server.

